# Effect of Initial Relative Density on Liquid-Phase Sintering Behaviors of Al Powder Using Al–Cu Eutectic Alloy Aid: In Situ Observations Using Tomography and Microscopy

**DOI:** 10.3390/ma18245499

**Published:** 2025-12-07

**Authors:** Ryotaro Kusunoki, Erika Matsumoto, Takeshi Higaki, Asuka Suzuki, Makoto Kobashi, Yukiko Ozaki, Masato Hoshino, Masayuki Uesugi

**Affiliations:** 1 Department of Materials Process Engineering, Graduate School of Engineering, Nagoya University, Furo-cho, Chikusa-ku, Nagoya 464-8603, Japan; 2 Joining & Welding Research Institute, Osaka University, 11-1 Mihogaoka, Osaka 567-0047, Japan; 3 Japan Synchrotron Radiation Research Institute, 1-1-1 Kouto, Sayo 679-5198, Japan

**Keywords:** liquid-phase sintering, aluminum powder, in situ observations, synchrotron radiation X-ray computed tomography, binder jetting

## Abstract

Aluminum (Al) powder with low sinterability is difficult to use in binder jetting (BJT) additive manufacturing, which involves sintering a metal powder after forming a green body. A liquid-phase sintering process for Al powder using Al–Cu eutectic alloy powder as a sintering aid has recently been developed. In this study, to clarify the applicability of liquid-phase sintering to BJT additive manufacturing, the effect of the initial relative density of green bodies (*ρ*_rel,0_ = 50–90%) on the final relative density was investigated. The final relative density was not significantly affected by *ρ*_rel,0_ and achieved 96–97% after sintering at 630 °C for 1800 s. However, pores are likely to remain in the sintered body with a high *ρ*_rel,0_ of 90%. In situ observations using synchrotron radiation X-ray computed tomography revealed that large pores were formed at the early sintering stage of the green body with *ρ*_rel,0_ of 90% and partially retained after sintering. By contrast, the green body with *ρ*_rel,0_ of 50% exhibited a significant rearrangement at the early sintering stage, promoting the densification. This study provides a deep understanding of liquid-phase sintering of Al powder, which is considered a suitable post-processing method for BJT additive manufacturing.

## 1. Introduction

Additive Manufacturing (AM) is a process of fabricating complex shapes by stacking materials layer by layer based on computer-aided design (CAD) data. The high design freedom of AM technologies is industrially significant not only because it can produce metal products with complex shapes or internal structures that cannot be realized with conventional technologies (machining, casting, forging, etc.), but also because it can reduce material waste [1].

Binder jetting (BJT) [2] is one type of AM process that can be applied to metals and alloys. A metal powder is spread on a building platform, and then a binder is injected into the powder. A green body is formed by repeating the powder spreading and binder injection. To impart sufficient strength to the BJT product, the green body is degreased and sintered at elevated temperatures. Compared with the beam-based metal AM processes (powder bed fusion (PBF) and directed energy deposition), BJT offers the advantages of high building speed and low equipment cost. A survey shows that pure iron and iron alloys are the most commonly studied metal materials for BJT technology, followed by nickel and its alloys, titanium and its alloys, and copper [3]. However, there has been little research on the application of aluminum (Al) and its alloys to BJT. Al is difficult to sinter because alumina film (Al_2_O_3_) covering the surface of Al particles inhibits sintering, making it difficult to produce dense Al products. Pressure-assisted sintering, which breaks the alumina film by the plastic deformation of Al particles, is effective for densifying Al products [4] but cannot be applied to BJT since BJT-built green bodies need to be densified while maintaining their complex shapes in the final sintering process.

Liquid-phase sintering is a promising technique that densifies Al products without pressure. The liquid phase, which accelerates the atomic diffusion during sintering, is generated through various routes, including the melting of elemental powders with a lower melting point than Al (Sn and Zn) [5], the eutectic reactions between Al and elemental powders (Cu, Si, Mg, and Zn) [5,6], and the semi-melting of Al alloy powders (Al–Si, Al–Si–Mg, and Al–Cu-Mg) (supersolidus liquid-phase sintering) [7]. Especially, supersolidus liquid-phase sintering, which heats Al alloy powder at a temperature between its solidus and liquidus temperatures, is widely studied as a sintering process for BJT because it can apply commercial Al–Si and Al–Si-Mg alloy powders used for PBF.

In another liquid-phase sintering process, Al powder is blended with eutectic Al alloy powders (Al–Si, Al–Cu, Al–Ca, and Al–Mg) as sintering aids and sintered at temperatures above their eutectic temperatures. The liquid phase generated by the melting of the eutectic alloy aids promotes the densification of Al products. It has been reported that a high relative density of 95–97% can be achieved by sintering an Al powder compact (relative density: ~75%) with Al–Si or Al–Cu eutectic alloy powders [8,9]. Thus, the liquid-phase sintering using eutectic alloy aids is also promising as a post-sintering process of BJT. Although the powder compacts with a relative density of approximately 75% can be densified by this liquid-phase sintering, the green bodies produced by BJT have a lower relative density of 40–60% [10]. In the case of liquid-phase sintering using Al and Cu elemental powders (eutectic reaction occurs at the interface between two powders), it is known that there is a positive correlation between the initial relative density and the final relative density [11]. Therefore, there is a possibility that the final relative density of the Al products sintered using eutectic alloy aid may decrease relative to the initial relative density. It is necessary to clarify the dependence of the initial relative density on the final relative density of Al products sintered using Al–Cu eutectic alloy powders.

In recent years, in situ observation of the sintering process has been actively conducted. For example, the solid-phase sintering process of alumina particles has been studied by in situ three-dimensional observation using synchrotron radiation X-ray computed tomography (CT) [12]. These studies can track changes in pore and particle shape over time, enabling a sophisticated understanding of the sintering process. Although synchrotron X-ray CT can observe the time evolution of the three-dimensional sintering process, it requires the rotation of the sample, making it difficult to capture the rapid progress of liquid-phase sintering. In the liquid-phase sintering process, in situ observations using scanning electron microscope (SEM) [13] and synchrotron radiation transmission X-rays [14] have been used for observing the sintering process microscopically (particle scale) and macroscopically (sintered body scale), respectively.

In this study, the effect of initial relative density (*ρ*_rel,0_) of the green body on the relative density of the Al products sintered by liquid-phase sintering using Al–Cu eutectic alloy aid was examined. Green bodies with different initial relative densities were sintered, and the relative density and microstructure of the sintered bodies were evaluated. These results indicate that pores remain within the sintered body at a high initial relative density. To elucidate the reason, multiple in situ observations using synchrotron radiation X-ray CT, SEM, and optical microscopy (OM) were performed to observe the liquid-phase sintering behavior. In particular, this is the first time that synchrotron radiation X-ray CT has been applied to observe liquid-phase sintering. Based on these results, the sequence of liquid-phase sintering with Al–Cu as the aid was discussed.

## 2. Experimental Procedure

### 2.1. Experiment Overview

As raw material powders, spherical Al (particle size: <45 µm, purity 99.99%, KOJUNDO CHEMICAL LABORATORY Co., Ltd., Saitama, Japan) and Al-17.3Cu (mol%) alloy powder (particle size: <45 µm, purity 99.99%, KOJUNDO CHEMICAL LABORATORY Co., Ltd., Saitama, Japan), prepared by gas atomization, were used as raw materials. The morphology and chemical compositions of the powders were reported in the literature [9]. These powders were mixed so that the average composition of the powder mixture was Al-1.9 mol% Cu [9]. Table 1 summarizes the conditions for sintering experiments and in situ observations. The green bodies were prepared by two different methods, and their relative densities were determined from their masses and volumes. The purpose of this study is to investigate fundamental liquid-phase sintering behaviors; therefore, the green bodies did not contain a binder, different from the BJT process. Firstly, the powder mixture was naturally filled into containers and tapped to obtain the green body with *ρ*_rel,0_ of approximately 50%. Secondly, the powder mixture was put into a graphite mold (inner diameter: 10 mm, outer diameter: 20 mm, and height: 50 mm) and heated up to 100 °C (temperature increase rate 0.34 °C/s) with a applied pressure of approximately 30 MPa while evacuating to approximately10 Pa using a spark plasma sintering apparatus (PLASMANKIT CSP-KIT-02121, S. S. Alloy Co., Ltd., Hiroshima, Japan) to produce a green body with *ρ*_rel,0_ of approximately 90%. Here, the reference phase for relative density was set to the α-Al phase containing 1.9 mol% Cu. Based on the atomic radius and weight of Al and Cu (radius, Al: 1.432 Å, Cu: 1.278 Å, weight, Al: 26.981, Cu: 63.546), the true density was estimated at 2.79 g‧cm^−3^, slightly higher than pure Al. Various experiments were conducted on the naturally filled and compacted bodies. For both samples, sintering at 630 °C for predetermined durations and subsequent characterization were carried out. The temperature and composition correspond to the α + Liquid two-phase region on the equilibrium phase diagram for the Al–Cu binary system, indicating that the liquid phase is stable during sintering. To understand the characteristic sintering behaviors observed in these experiments, in situ observations were performed: in situ observation using an optical microscope for naturally filled samples with *ρ*_rel,0_ = 50% and in situ observations using synchrotron radiation X-ray CT and an SEM were conducted for the compacts with *ρ*_rel,0_ = 90%. The details of these experiments are described below.

### 2.2. Investigation of Sintering Behaviors of Green Bodies with Different Initial Relative Densities

To investigate the sintering behaviors of the green bodies with different relative densities, the naturally filled powder or powder compact was sintered using a high-frequency induction furnace (VMF-I-I1C, DIAVAC LIMITED, Chiba, Japan). When the green body with *ρ*_rel,0_ of approximately 50% was sintered, the powder mixture was naturally filled into a 12 mm alumina tube (Nikkato SSA-S tungsten T2: Al_2_O_3_) and tapped. When the powder compact with *ρ*_rel,0_ of approximately 90% was sintered, 2.5 g of Al_2_O_3_ powder (particle size: 150 μm) was placed into the alumina crucible, on which the powder compact was placed. The alumina crucible was put into a graphite mold and set inside the induction furnace. The furnace interior was evacuated to approximately 4 Pa and filled with Ar gas with a pressure of approximately 0.05 MPa. The green bodies were heated to approximately 630 °C and held for 0, 120, 300, 720, and 1800 s, then cooled to ambient temperature by turning off the power of the induction heating. The temperature was monitored and controlled by a thermocouple placed near the green bodies. The detailed setup for sintering in the furnace was given elsewhere [9]. The relative density of the sintered bodies was evaluated by the Archimedes method. The samples were immersed in an epoxy resin, mechanically polished using SiC papers, buffed using 3 μm and 1 μm diamond slurries, and finished with a colloidal silica. The microstructure was observed using an SEM (JSM-6610A, JEOL, Tokyo, Japan). Elemental distributions were analyzed by energy-dispersive X-ray spectroscopy (EDS) analysis. 

### 2.3. In Situ X-Ray CT Observation

**Figure 1:**
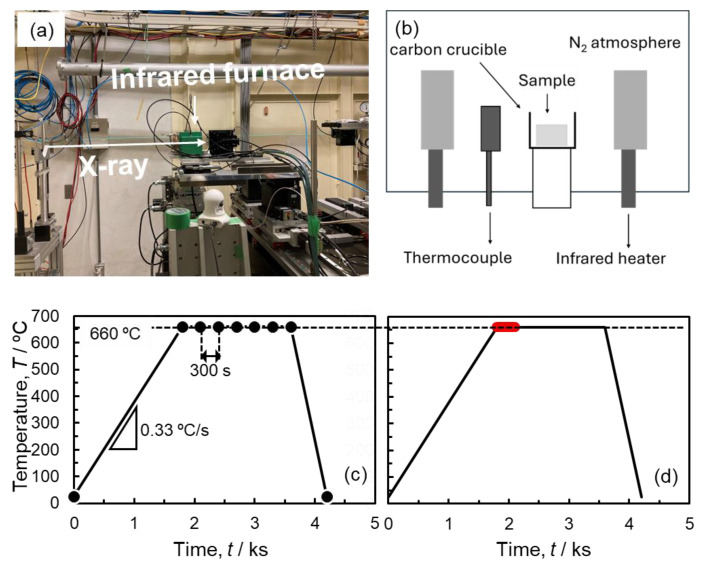
(**a**) Photographs showing the setup of in situ synchrotron X-ray computed tomography (CT) observation, (**b**) schematic illustration inside the infrared heating furnace, and (**c**) batch and (**d**) continuous imaging patterns shown on the temperature profile during the heating. The images were taken at the times indicated by the black dots in (**c**) and the periods indicated by the red shapes in (**d**).

To observe the sintering behavior of the powder compact with *ρ*_rel,0_ of approximately 90%, synchrotron X-ray CT was performed using the beamline BL20B2 of SPring-8 (Hyogo, Japan), a large synchrotron radiation facility in Japan. The powder compact was cut into small pieces with a size of 1 mm × 1 mm × 2 mm. A piece was put into a carbon crucible, and the carbon crucible was set in the infrared heating apparatus through a window that is X-ray transmittable. Figure 1a,b shows (a) the setup of in situ synchrotron X-ray CT observation and (b) a schematic illustration inside the infrared heating furnace. The temperature was monitored and controlled using a thermocouple near the crucible (Figure 1b). Because the thermocouple was set outside the crucible, it was necessary to calibrate the measured temperature against the sample temperature. For calibration, high-purity Al (purity: 99.99%) and Al-17.3Cu eutectic alloy bulks with known melting temperatures (660 °C and 548 °C) were heated using the infrared furnace while observing these samples by transmission X-ray at the beamline. The high-purity Al and Al-17.3Cu alloy were melted at measured temperatures of approximately 690 °C and 570 °C, respectively. Thus, when the powder compact was sintered in the infrared furnace, the thermocouple temperature was controlled at 660 °C so that the sample temperature was approximately 630 °C. The atmosphere during sintering was a N_2_ gas flow. The synchrotron radiation energy was 40 keV, and the resolution (pixel size) was 1.92 μm.

Figure 1c,d shows the temperature histories and imaging patterns of the In Situ observations. For the three-dimensional observations, the sample needs to be rotated by 180°, which takes 30 s. Before heating, the pieces cut from the powder compact were observed in three dimensions. When the thermocouple temperature reached 660 °C, the sample was rotated and irradiated with X-rays to capture the three-dimensional image. Subsequently, two types of imaging were carried out, namely: (i) batch imaging (Figure 1c) and (ii) continuous imaging (Figure 1d). In the batch imaging, the sample rotation and X-ray irradiation were carried out every 300 s from the start of holding at 660 °C to 1800 s to roughly capture the sequence of the three-dimensional sintering process. In addition, the imaging was carried out after cooling to the ambient temperature. As a result, it was found that the sintering phenomena rapidly proceeded during the early stage of holding (until 300 s). To capture the rapid progress of the sintering phenomena in the early stages of sintering, the sample was kept rotating and continuously irradiated with X-rays for 210 s. Continuous rotation allows for the extraction and reconstruction of a transmission image dataset taken during an arbitrary 180° rotation, presumably making it possible to capture the rapid sintering progress. The transmission images were reconstructed to cross-sectional image stacks using homemade software (ct-rec, http://www-bl20.spring8.or.jp/xct/, accessed date: 6 December 2025) at SPring-8. The cross-sectional image stacks were imported into three-dimensional structural analysis software (VG-STUDIO MAX 2023.4, Volume Graphics GmbH, Heidelberg, Germany) to construct three-dimensional images and to focus on the morphology of pores. Image analysis software (ImageJ 1,54f, National Institutes of Health, Bethesda, MD, USA) was used to binarize the cross-sectional images and calculate the porosity and pore size.

### 2.4. In Situ SEM Observation

**Figure 2:**
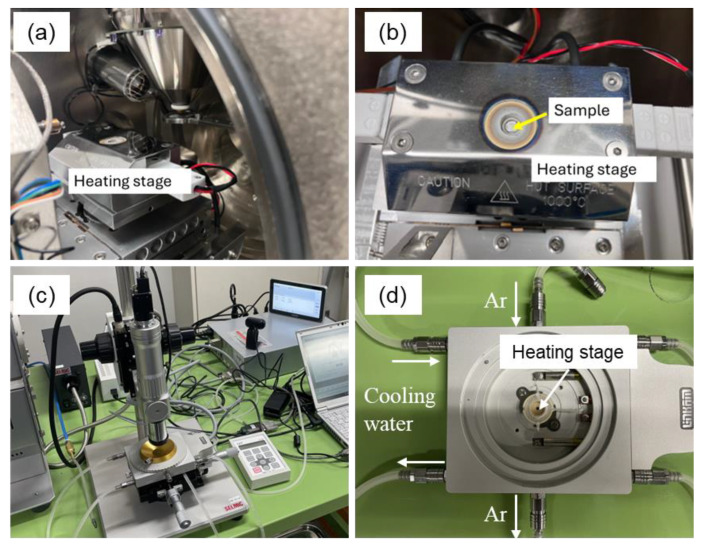
Photographs showing (**a**,**c**) setup and (**b**,**d**) sample stage of in situ observations using (**a**,**b**) scanning electron and (**c**,**d**) optical microscope.

To support the In Situ X-ray CT observations, the melting and infiltration behaviors of Al–Cu aid in the green body with *ρ*_rel,0_ of approximately 90% were observed in situ using an environmental SEM (Quanta FEG250, Thermo Fisher Scientific K.K., Waltham, MA, USA). Figure 2a,b shows the photographs of (a) the setup in the SEM chamber and (b) the sample heating stage. The atmosphere in the SEM was an Ar gas flow, and its pressure was 50 Pa. The temperature increased at a rate of 0.5 °C/s and was kept at 630 °C. The image acquisition interval was 2 s.

### 2.5. In Situ Optical Microscopy

The sintering behaviors of the powder mixture with *ρ*_rel,0_ of approximately 50% was observed in situ using an optical microscope (SE-2000WR-3D, SELMIC, Shiga, Japan) combined with a heating stage (10016, JAPAN HIGH TECH CO., LTD, Fukuoka, Japan). Figure 2c,d shows the photograph of (c) the experimental setup and (d) the heating stage. The powder mixture was placed in a sapphire circular container with an outer diameter of 6.5 mm, inner diameter of 5.5 mm, and height of 1.0 mm, and then tapped. The container was set on the sample stage shown in Figure 2d. The Ar gas flow was applied to prevent further oxidation of Al powder during heating. The heating stage was heated at a rate of 0.5 °C/s and held at 630 °C for 1800 s. The sintering behavior during heating was captured by the optical microscope.

## 3. Results

### 3.1. Sintering Behaviors of Green Bodies with Different Initial Relative Densities

**Figure 3:**
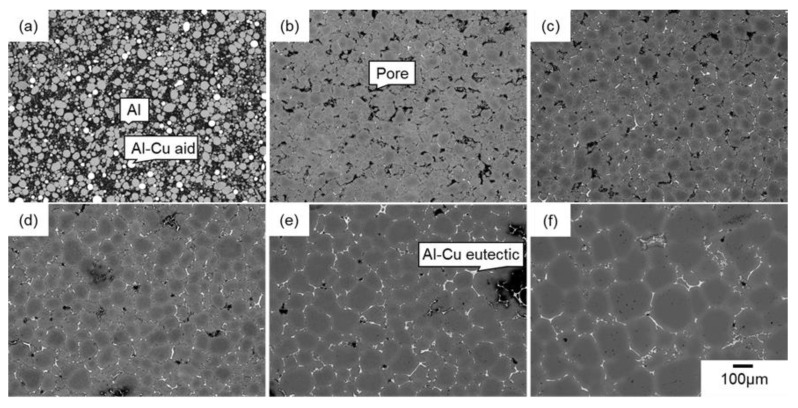
SEM images of (**a**) powder mixture with an initial relative density of approximately 50% and samples sintered at 630 °C for (**b**) 0 s, (**c**) 120 s, (**d**) 300 s, (**e**) 720 s, (**f**) 1800 s.

**Figure 4:**
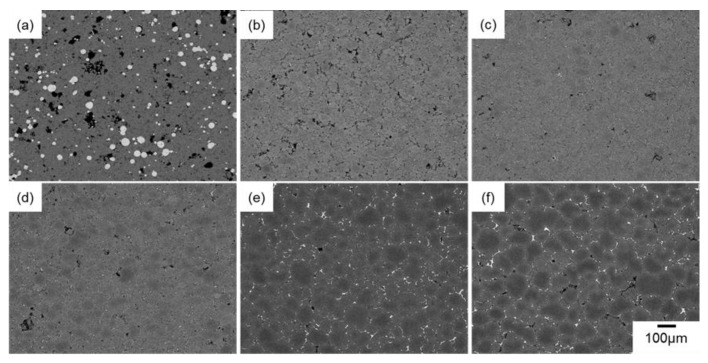
SEM images of (**a**) powder compact with an initial relative density of approximately 90% and samples sintered at 630 °C for (**b**) 0 s, (**c**) 120 s, (**d**) 300 s, (**e**) 720 s, (**f**) 1800 s.

Figure 3 and Figure 4 present SEM images of the naturally filled powder mixture (Figure 3a) with *ρ*_rel,0_ of 50%, powder compact with *ρ*_rel,0_ of approximately 90% (Figure 4a), and their sintering behaviors at 630 °C (Figure 3 and Figure 4b–f). In Figure 3a and Figure 4a, the darkest, middle-brightness, and brightest parts indicate pores, Al, and Al–Cu aid, respectively. In Figure 3a, both Al and Al–Cu particles are clearly visible, whereas Al particles are bonded and cannot be seen as particles in Figure 4a. This indicates that Al particles were pre-sintered by the process to prepare the powder compact. In Figure 3 and Figure 4b–f, the darkest, and intermediately bright, and the brightest areas are pores, α-Al phase, and θ-Al_2_Cu phase. The θ phase was formed by the eutectic reaction, Liquid→α + θ, during cooling after sintering, indicating that the liquid phase existed at the brightest areas during sintering. The densification rapidly progressed by heating the naturally filled powder mixture with *ρ*_rel_,_0_ of approximately 50% to 630 °C (Figure 3a,b). Holding at 630 °C gradually densified the sample and promoted the grain growth (Figure 3b–f). These observations indicate that a highly dense sample can be obtained by liquid-phase sintering with Al–Cu as an aid, even when the initial relative density is as low as 50%. When the powder compact with *ρ*_rel,0_ of 90% was sintered, a slight densification occurred at 630 °C. It is noted that more pores were observed in the sample with *ρ*_rel,0_ of 90% than in that with 50% sintering at 630 °C for 1800 s (Figure 3f and Figure 4f). In addition, grain growth was less pronounced, and the θ phase was discontinuously distributed in the sample with an initial relative density of 90%.

**Figure 5:**
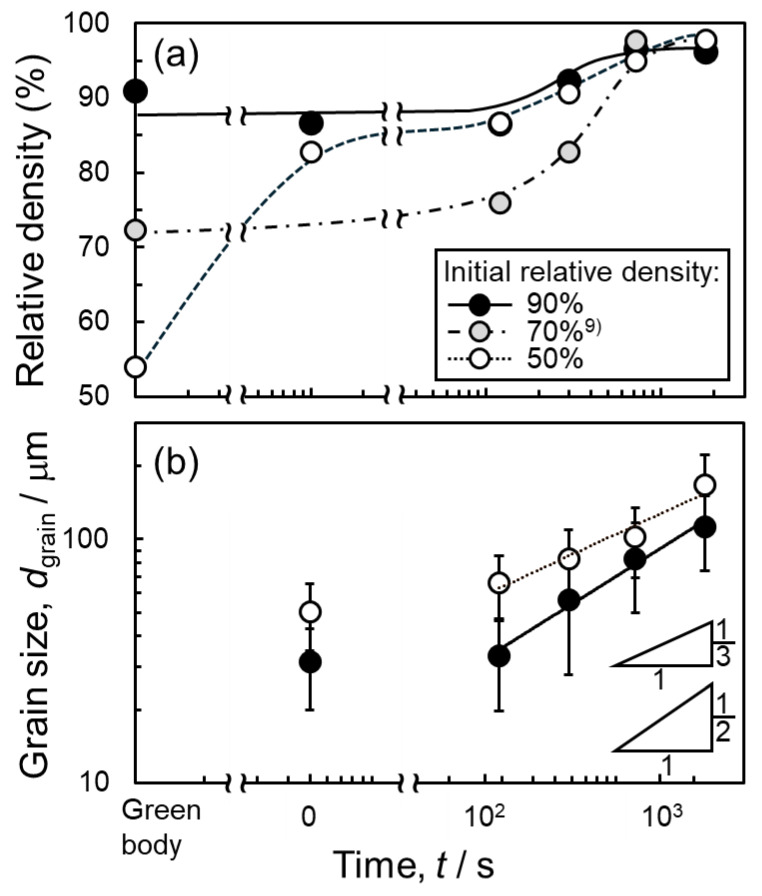
Changes in (**a**) relative density and (**b**) grain size as a function of sintering time at 630 °C. For comparison, the relative density of the sample sintered from the green body with an initial relative density of 70% [9] was also shown in (**a**).

Figure 5 shows the change in relative density and grain size of the samples with different initial relative densities as a function of sintering time. In Figure 5a, the change in the relative density of the sample with an initial relative density of approximately 70% reported in the literature [9] was also shown for comparison. The relative density of all the samples reached approximately 96–97% after sintering for 1800 s. The results show that the final relative density does not vary much with the initial relative density during liquid-phase sintering with the Al–Cu aid. Compared to samples sintered with *ρ*_rel,0_ of 50% and 70%, the sample with *ρ*_rel,0_ of 90% reached a slightly lower relative density of 96%, which was in accordance with frequently observed pores in Figure 4f. These trends were different from the trend reported in the literature on liquid-phase sintering using elemental powders [11]. It was also found that the relative density of the sample with *ρ*_rel,0_ of 50% increased rapidly in the early stages of sintering, which was in good agreement with the SEM observation shown in Figure 3a,b. Densification at the early stage of sintering was suppressed in the powder compacts with *ρ*_rel,0_ of 70% and 90%. As a result, the relative density of the sample with *ρ*_rel,0_ of 50% was higher than that of the sample with *ρ*_rel,0_ of 70% up to 300 s. Only the sample with *ρ*_rel,0_ of 50% was naturally filled in the crucible and not compressed prior to the sintering. The compression would suppress densification at the early stage of sintering.

**Figure 6:**
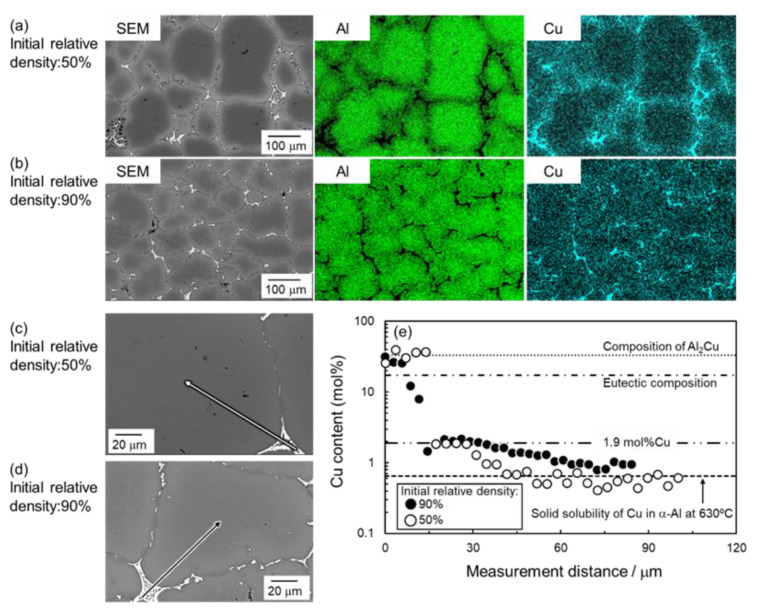
SEM images and the corresponding EDS element maps of Al and Cu in the samples sintered at 630 °C for 1800 s using the green bodies with relative densities of (**a**,**c**) 50% and (**b**,**d**) 90%. (**e**) EDS line analysis results along with the arrows shown in (**c**,**d**).

Both samples with *ρ*_rel,0_ of 50% and 90% showed grain growth, and their grain size (*D*_grain_) reached more than 100 μm. It is known that the grain growth in the sintering was expressed as a formula, *D*_grain_ − *D*_grain, 0_ = *kt^n^*, where *D*_grain_, _0_ is an initial grain size, *k* is the rate constant, *t* is the sintering time, and *n* is the exponent factor [15]. Especially, *n* reflects the rate-limiting process of the grain growth and can be quantified by the slope of the line shown in Figure 5b. The sample with *ρ*_rel,0_ of 50% showed a slope close to 1/3, whereas the sample with *ρ*_rel,0_ of 90% showed a slope close to 1/2. The slope of 1/3 and 1/2 indicates that Ostwald ripening (dissolution of elements from small particles into the liquid phase and reprecipitation on large particles) and grain boundary migration limit the grain growth [16,17]. Thus, the rate-limiting process of grain growth during liquid-phase sintering using Al–Cu aid would depend on the initial relative density. Figure 6a,b shows the elemental distribution of the sample sintered at 630 °C for 1800s using green bodies with *ρ*_rel,0_ of (a) 50% and (b) 90%. Cu was concentrated on the θ phase and α grain boundaries. To further investigate the diffusion of Cu during the sintering, the EDS line analysis was carried out from the θ phase (eutectic area) to the α grain interior. At around the center of the grains, both sintered samples with *ρ*_rel,0_ of 50% and 90% exhibited a Cu concentration close to the equilibrium Cu concentration in the α-phase at 630 °C. This indicates that both samples reached the equilibrium state at 630 °C after sintering for 1800 s. The Cu concentration gradually increased toward the grain boundaries, which was caused by the growth of the α phase (migration of the solid/liquid interface) during the cooling from the sintering temperature of 630 °C [9]. At 630 °C, α and liquid phases coexisted based on the equilibrium phase diagram for the Al–Cu binary system. During the cooling, the solid/liquid interface migrated under a local equilibrium at the interface. As a result, the Cu element was partitioned into the α phase following the solidus line of the phase diagram, and its concentration increased toward the grain boundaries (A more detailed mechanism is given elsewhere [9]). At α grain boundaries, a high Cu concentration close to the composition of the θ phase was detected since the liquid phase finally decomposed into α + θ phases during the solidification. These results were in accordance with the sample sintered from the green body with of *ρ*_rel,0_ of 70% [9]. Thus, the elemental diffusion during sintering was not affected by the initial relative density.

### 3.2. In Situ Synchrotron Radiation X-Ray CT Observation

**Figure 7:**
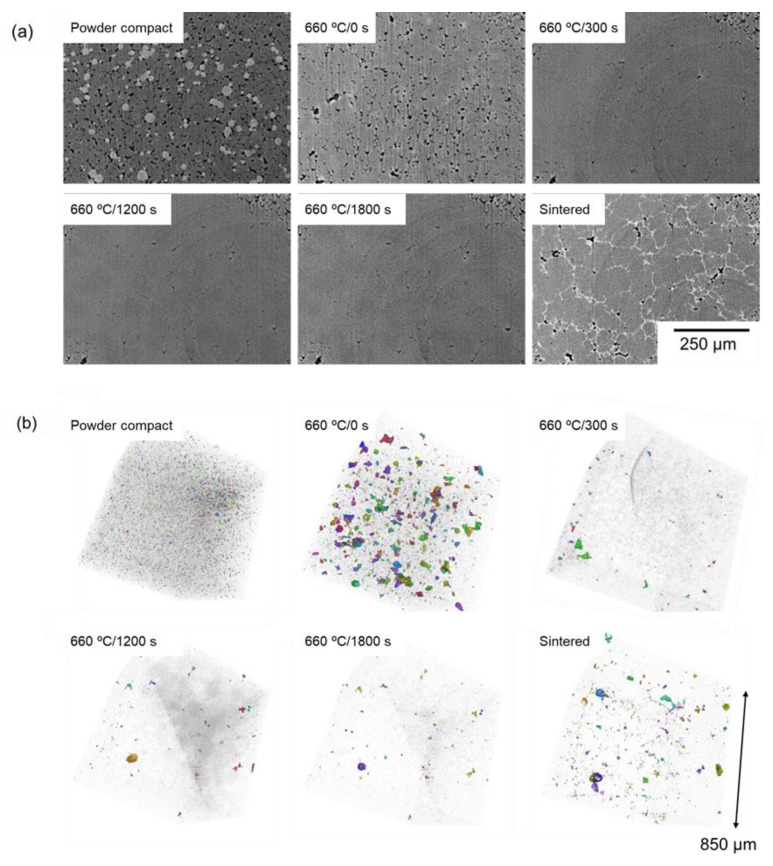
Synchrotron radiation X-ray CT images of (**a**) cross-sectional areas and (**b**) three-dimensional pore distributions of the powder compacts with an initial relative density of 90% and their sintering sequence at 660 °C.

Figure 7a shows the synchrotron radiation X-ray CT images showing the cross-sections taken initially and every 300 s during sintering of the compact. In X-ray CT images, the area appears brighter as the absorption and scattering of X-rays occur significantly. In addition, X-ray absorption and scattering are more pronounced in areas with higher density. Therefore, the spherical bright particles, intermediately bright areas, and dark areas are Al–Cu aids, Al, and pores, respectively. The CT images taken during sintering at 630 °C have two distinct contrasts between pores and the matrix. Unfortunately, the solid and liquid phases could not be distinguished in the images. This might be due to the small difference in the density between the solid and liquid phases. In general, Al melt has a lower density than Al solid [18]. However, in this system, Cu, which is a heavier element than Al, is more partitioned into the liquid phase than into the solid phase at the sintering temperature. These effects might cancel each other out, resulting in a small contrast difference between the solid and liquid phases. This was supported by the image after cooling (denoted as “sintered” in Figure 7a. In this image, bright contrasts, which would correspond to the θ phase, are visible at the grain boundary. After cooling, both the α and θ phases are solid, making them distinguishable by their density difference. Although distinguishing solid and liquid phases needs to be realized in future work, clearly visible pores were focused on in this study and extracted in the three-dimensional images shown in Figure 7b. Fine pores were present in the powder compact in the three-dimensional image. Interestingly, numerous large pores were generated when the temperature reached 660 °C (660 °C/0 s) and rapidly disappeared within 300 s. The rapid pore formation and disappearance were not captured in Figure 4 and could be visible through in situ observations. After 300 s, there is a slight change in the pore distribution and morphology. Comparing images of 660 °C/1800 s and the sintered body, the pores were formed during the cooling process. Based on the cross-sectional images, the pores were formed around the θ phase. The θ phase region corresponded to the α/θ eutectic structure (Figure 3 and Figure 4) and corresponded to the liquid phase at the sintering temperature. Therefore, the pores were formed during the cooling process in the region where the liquid phase existed. This phenomenon would be explained by the previous study [9]. In the previous study, numerous pores were observed in the sample sintered using Al–Mg aid, and Mg was homogeneously distributed in the sample, even though the sintering was carried out α + Liquid two-phase region. Mg has a large solubility limit in the α phase, and would be dissolved into the α phase during the cooling process. Then, the volume increment of the α phase during the cooling would not compensate for the volume disappearance of the liquid phase, forming the pores in the region where the liquid phase existed. Cu has a smaller solid solubility limit in the α phase than Mg, and its distribution was not homogenized during the cooling. However, similar phenomena would occur during the cooling process, resulting in the formation of pores around the θ phase.

**Figure 8:**
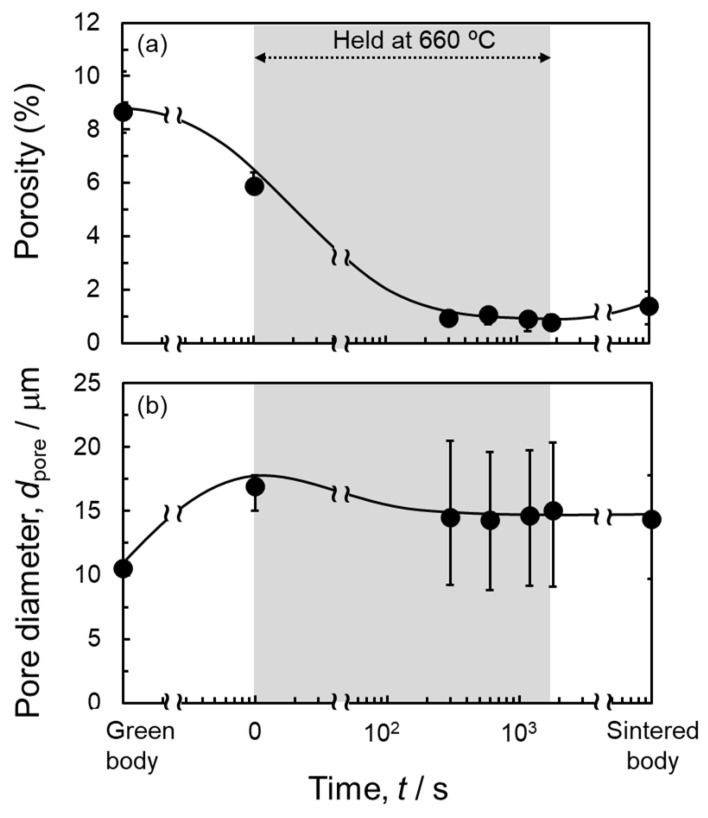
Changes in (**a**) porosity and (**b**) pore diameter during the sintering process of powder compacts with an initial relative density of 90% in situ observed by synchrotron X-ray CT.

Figure 8 shows the change in porosity and average pore diameter analyzed using a cross-sectional CT image as a function of sintering time. The error bars indicate variations in porosity and average pore diameter measured at different cross-sections and do not mean the variations between multiple samples (therefore, the error in porosity is relatively small). The porosity of the green body was approximately 8.7%, which was in good accordance with the measured relative density of approximately 91%. The average pore diameter of the green body was approximately 10 μm. The porosity was decreased to 5.9% as the thermocouple temperature increased to 660 °C. Then, the average pore diameter increased to approximately 17 μm due to the formation of large pores in Figure 7b. The porosity decreased rapidly in the early stages of sintering and remained almost constant after 120 s until the end of the sintering temperature hold. The average pore diameter decreased but remained higher than that of the green body.

**Figure 9:**
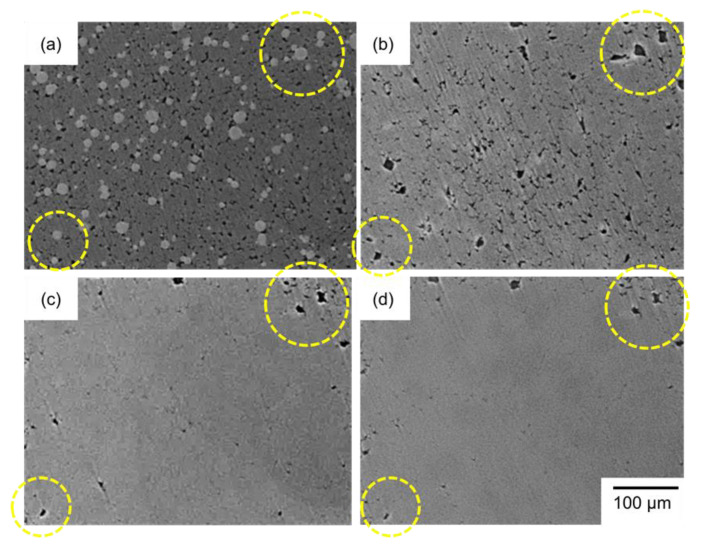
Cross-sectional synchrotron radiation X-ray CT image at the same field of view; (**a**) powder compact with an initial relative density of 90% and its sintering sequence at 660 °C for (**b**) 0 s, (**c**) 300 s, (**d**) 1800 s.

To identify the reason why the large pores were formed at the early stage of sintering, the cross-sectional X-ray CT images with an identical field of view were carefully checked and are shown in Figure 9. Focusing on the location where coarse pores were formed at 660 °C/0 s (yellow circle in Figure 9b), the Al–Cu aid (bright particles) existed in the green body (yellow circle in Figure 9a), indicating that the large pores would be formed by the melting and infiltration of Al–Cu aid into fine pores. In addition, some of the large pores were retained even after the sintering for 1800 s (Figure 9c,d), suggesting that the formation of large pores at the early stage of sintering inhibited the densification at the late sintering stage.

**Figure 10:**
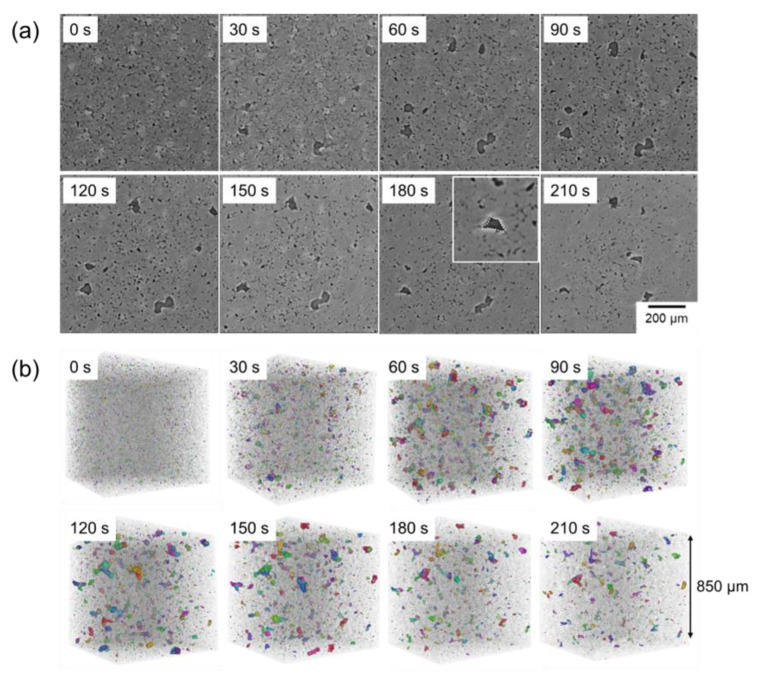
Synchrotron radiation X-ray CT images of (**a**) cross-sections and (**b**) three-dimensional pore distributions of the powder compact with an initial relative density of 90% obtained by continuous imaging at 660 °C for 0 s, 30 s, 60 s, 90 s, 120 s, 150 s, 180 s, and 210 s. In the cross-sectional image at 180 s, the curvature at the solid/pore interface is also shown.

As shown in Figure 7, the formation and disappearance of large pores rapidly occurred during the early stage of sintering. Since imaging every 300 s could not capture its details, continuous imaging was carried out, with the sample kept rotating and irradiated with X-rays. In these figures, reconstruction was carried out using transmission images taken every 30 s, as shown in Figure 10. In this experiment, large pores were not formed when the thermocouple temperature reached 660 °C, which was different from the results shown in Figure 7. There would be some variation in the timing of the formation of large pores. However, a continuous sequence of occurrence, shrinkage, and disappearance of large pores was captured by the continuous imaging. When the large pores were shrunk, the curvature of the pores was convex toward the pore side (shown in the cross-section at 180 s), suggesting that the pore shrinkage might be caused by the grain growth toward the pore side, although further observations to make individual grains visible need to be carried out using not only the transmission X-ray but also the diffraction X-ray.

### 3.3. In Situ Microscopy Observations

**Figure 11:**
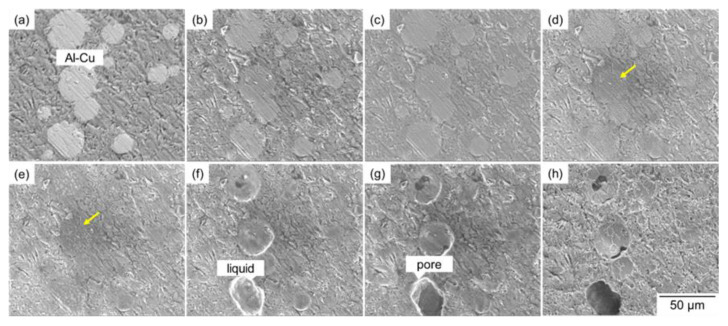
SEM images showing (**a**) powder compact with an initial relative density of 90%, (**h**) its sintered body cooled to ambient temperature after sintering at 630 °C for 1800 s. (**b–f**) SEM images showing the microstructural change observed in situ at (**b**) 30 °C, (**c**) 550 °C, and 630 °C for (**d**) 0 s, (**e**) 120 s, (**f**) 180 s, and (**g**) 900 s. At (**c**,**d**), the microstructure in the Al-Cu aid is visible, as indicated by the yellow arrows.

In Section 3.2, the large pores were formed at the early stage of sintering and originated from the Al–Cu aid. The formation of the large pores would be due to the infiltration of the Al–Cu melt into fine pores. However, since solid and liquid phases were not distinguished in the synchrotron X-ray CT, the infiltration of Al–Cu melt could be directly observed. Therefore, to support the synchrotron X-ray CT observations, in situ SEM observations were carried out on the powder compact with *ρ*_rel,0_ of 90%, as shown in Figure 11 (see also the movie shown in the Supplementary Material Video S1). Since rapid scanning was required during heating to capture rapid phenomena, the SEM images during heating were relatively noisy (Figure 11b–g), whereas clearer images were taken by slow scanning before heating and after cooling (Figure 11a,h). Comparing the images before heating and after cooling, concavity was formed at the locations where the Al–Cu aid was present. During heating, the microstructure in the Al–Cu aid became visible due to its coarsening (Figure 11c–e). Subsequently, the Al–Cu aid was melted (Figure 11f) and infiltrated into the sample interior (Figure 11g). The concavity formed by the infiltration of Al–Cu melt remained after cooling (Figure 11h) since the liquid phase assisting sintering was absent from the observation surface. These observations directly indicated that the formation of large pores at the early stage of sintering (Figure 7) was caused by the melting and infiltration of Al–Cu aid. In these observations, the coarsening of microstructure and the melting of Al–Cu aid occurred after starting to hold the temperature at 630 °C, although the eutectic temperature of Al–Cu aid is 548 °C. This was caused by the difference between the sample temperature and the temperature measured by the thermocouple set below the sample stage.

**Figure 12:**
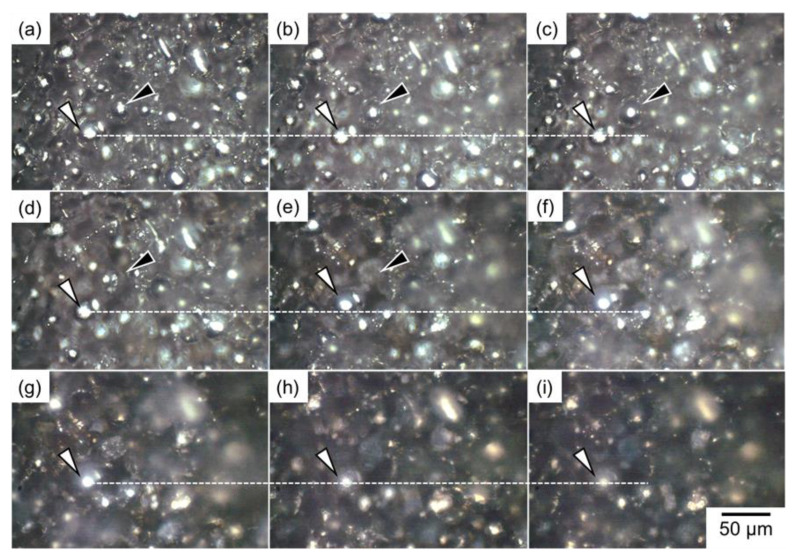
OM image of (**a**) powder mixture with an initial relative density of approximately 50% and (**b**–**i**) its microstructural change observed in situ at (**b**) 550 °C, (**c**) 600 °C, and 630 °C for (**d**) 0 s, (**e**) 60 s, (**f**) 120 s, (**g**) 300 s, (**h**) 720 s, and (**i**) 1800 s. The particle indicated by the black triangle is melted at (**d**) and infiltrated at (**e**). The particle indicated by the white triangle moves upward (see the white dashed line as the guideline).

To investigate the reason why the rapid densification of a naturally filled powder mixture with *ρ*_rel,0_ of 50%, in situ OM observations were carried out and shown in Figure 12 (see also the video shown in Supplementary Material Video S2). A significant rearrangement was observed at a temperature of around 600 °C and continued for approximately 370 s (310 s after being held at 630 °C). For example, the surface of the particle indicated by the black triangle in Figure 12 became smooth during heating to 630 °C (Figure 12a–c) due to its melting (This particle was the Al–Cu aid). After holding at 630 °C, the particle melted and infiltrated inside the sample (Figure 12c–d). Then, the particle indicated by the white arrow moved upward in the image (Figure 12d–f; see the white dashed lines as guidelines). After that, particle arrangements remained almost unchanged, although the brightness of the sample became darker due to the surface oxidation (Figure 12g–i).

## 4. Discussion

In this study, the effect of initial relative density (*ρ*_rel,0_) on the final relative density of the sintered body through the liquid-phase sintering process using Al–Cu eutectic alloy aid was investigated. The process enabled the fabrication of dense samples with a final relative density of 96–97% by sintering at 630 °C for 1800 s regardless of the initial relative density in the range of 50–90%. Especially, naturally filled powder with *ρ*_rel,0_ of approximately 50% showed a rapid densification at the early stage of sintering (Figure 3a,b and Figure 5a). The characteristic of the process is advantageous as the sintering process of BJT additive manufacturing, which requires the densification of the green body with a low relative density of 40–60%. In this sintering process, the pores tended to remain in the sintered body when the initial relative density was as high as 90% (Figure 4f and Figure 5a). The initial relative density dependence of the sintering behavior in this process is discussed here based on in situ observations.

**Figure 13:**
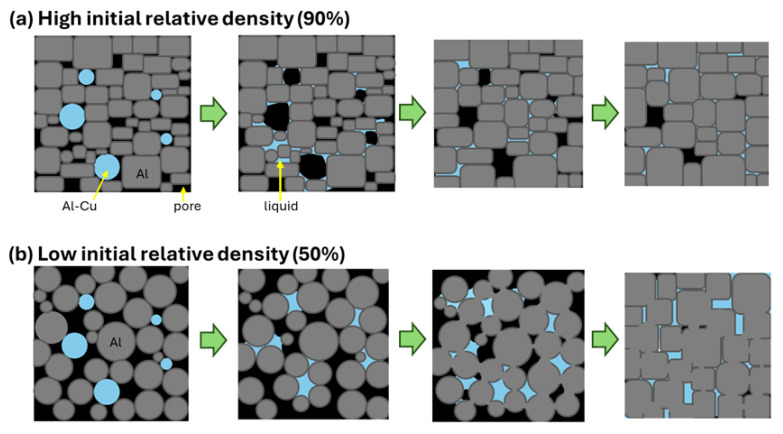
Schematic illustrations showing the sintering sequence of the green bodies with (**a**) a high initial relative density of 50% and (**b**) a low initial relative density of 90%.

Figure 13 schematically illustrates the sintering behaviors of the green bodies with *ρ*_rel,0_ of (a) 90% and (b) 50%. At the early stage of sintering of powder compacts with *ρ*_rel,0_ of 90% (Figure 13a), large pores were formed by infiltration of the Al–Cu alloy melt into fine pores, as observed in Figure 7, Figure 9,and Figure 11. It is known that this phenomenon occurs when the aid has a large size, and the porosity of the green body is low [19,20]. The formation of large pores delayed densification at the early sintering stage, presumably causing almost constant relative density at the early stage of sintering of samples with *ρ*_rel, 0_ of 70% and 90%(Figure 5a). These two samples were compressed before the sintering. The compression would partially fix the arrangement of particles in the green body, suppressing the rearrangement and its associated densification at the early sintering stage. Almost all large pores disappeared during grain growth (Figure 7, Figure 10 and Figure 13a), but some large pores were retained even after sintering for 1800 s (Figure 9), presumably due to the formation of closed pores. It is known that closed pores are difficult to remove if the inert gas (N_2_ and Ar) is trapped inside the pores [21]. In this study, sintering was carried out under a N_2_ gas flow or an Ar gas atmosphere. The closed pores trapping these gases would be less likely to disappear. The formation of large pores through Al–Cu melt infiltration into fine pores would increase the probability of forming closed pores, tending to retain the pores in the sintered body (Figure 4f and Figure 13a). By contrast, a significant rearrangement (Figure 13b) occurred at the early stage of sintering of the naturally filled powder with *ρ*_rel,0_ of 50% (Figure 12 and Figure S2), causing a rapid densification (Figure 5a). The rearrangement of particles instead of melt infiltration did not form large pores and was less likely to form closed pores, leading to highly dense sintered products.

In the case of liquid-phase sintering of Al and Cu elemental powders, the final relative density positively correlates with the initial relative density [11]. By contrast, the final relative density was not significantly affected by initial relative density in the case of liquid-phase sintering using Al–Cu aid. This would be related to the difference in the liquid phase formation pathway. In the case of the liquid-phase sintering using the elemental powders, interdiffusion at the interface between Al and Cu particles partially reduces the melting temperature and forms a liquid phase [22]. To increase the contact area between particles and promote the formation of the liquid phase, a high initial relative density achieved through compaction is required. In contrast, Al–Cu aid is composed of a fine α/θ two-phase eutectic structure [9]. This fine eutectic structure allows the liquid phase to form with a slight interdiffusion, as observed in the coarsening of the eutectic microstructure and the subsequent melting (Figure 12d–f). As a result, a sufficient amount of liquid phase would be supplied, making it possible to achieve a high relative density regardless of the initial relative density.

The infiltration of Al–Cu melts into fine pores and the associated formation of large pores, observed in the green body with *ρ*_rel,0_ of 90%, would be driven by the capillary force. In addition, the rearrangement of Al powder and its associated densification at the early stage of sintering of the powder mixture with *ρ*_rel,0_ of 50% would be caused by the capillary force of wetting liquid. These results consistently showed that the Al–Cu melt exhibited good wettability to Al powder. The good wettability allowed the capillary force to act in the direction of drawing the liquid phase into fine pores and bringing the powder particles closer together. The surface of Al powder is covered with alumina film (amorphous structure in the case of naturally formed alumina) [23]. Therefore, it is suggested that the Al–Cu melt could have good wettability toward the alumina film. However, it is well known that Al and its alloy melts usually exhibit a poor wettability to alumina around the melting temperature of Al (the contact angle is higher than 90°) [24,25]. The addition of Cu into the Al melt also has a slight effect on the poor wettability [26]. The good wettability of Al–Cu melt to Al powder, which is suggested in this study, seems to contradict these reports. A possible reason is presented below. At first, a large difference in thermal expansion rate between Al and alumina would generate tensile stress at elevated temperatures and form cracks in the alumina film [13,27,28]. Then, the bare Al surface could be partially and temporarily exposed. It is reported that an Al-coated Al_2_O_3_ plate exhibited a low contact angle of approximately 12° with molten Al at 700 °C [29]. Thus, if molten Al or its alloy contacts a bare Al surface, it would wet the powder surface. This hypothesis is also supported by the rapid progress of the liquid-phase sintering process. Sintering and associated elemental diffusion were sufficient to densify the sintered body at 630 °C for 1800 s. If the Al powder surface is completely covered with alumina, sintering would be rate-limited by the elemental diffusion in alumina with a high melting point, regardless of the presence or absence of a liquid phase. For example, the diffusion coefficient of Al in polycrystalline α-Al_2_O_3_ at 630 °C can be estimated at 7.3 × 10^−31^ m^2^/s, based on the activation energy (477 kJ/mol) and frequency factor reported (2.8 × 10^−3^ m^2^/s) at a temperature range of 1670–1905 °C [30,31]. Then, the diffusion distance during 1800 s is estimated at 5.1 × 10^−8^ μm, indicating the difficulty of diffusion and sintering via the alumina film. Therefore, the rapid sintering process also suggested that the bare Al surface was in direct contact with the liquid phase. However, nano-scale phenomena were not observed in this study, and further investigations, including in situ observations of the oxide film using transmission electron microscopy [32], need to be carried out in future studies.

This study showed that the final relative density of the sintered body obtained via liquid-phase sintering using the Al–Cu aid was slightly affected by the initial relative density. In particular, a significant arrangement at the early stage of sintering enabled the fabrication of a densely sintered body with a final relative density over 97%, even when the initial relative density was approximately 50% (it is in the range of the relative density of the green body fabricated by BJT additive manufacturing). Thus, this study implies that the liquid-phase sintering process using Al–Cu eutectic alloy powder can be applied to the densification process of the green body fabricated by BJT additive manufacturing. The green body fabricated by the BJT process needs to be sintered through this process in future studies. The BJT-produced green bodies contain a binder, whereas the green bodies without a binder were sintered in this study. Although the binder is thermally or chemically removed before sintering, the remaining binder would exist and inhibit the sintering [2]. Although studies have examined the use of Cu powders in BJT [33] and have investigated binder formulations and degreasing strategies for other metal powders [34], no research has specifically explored the interactions between Al powder or Al–Cu aid and binders. Liquid-phase sintering behaviors of the BJT-produced green bodies using the Al–Cu aid need to be clarified in future studies.

## 5. Conclusions

In this study, the effect of initial relative density on the sintering behavior of Al powder via liquid-phase sintering using Al–Cu aid was investigated to clarify the applicability of this process to the densification of green bodies fabricated by binder jetting (BJT) additive manufacturing. To capture the rapid progress of liquid-phase sintering, various in situ observations using X-ray and microscopy were conducted. The following findings were obtained:

- Even when the initial relative density was varied in the range of 50–90%, the liquid-phase sintering at 630 °C for 1800 s achieved a high final relative density of 96–97%. The final relative density was less affected by the initial relative density in the case of this liquid-phase sintering.
- The pores tended to remain in the sample sintered using a dense green body with an initial relative density of approximately 90%. In situ observation using synchrotron radiation X-ray computed tomography (CT) revealed that large pores formed early in sintering, and some of these pores remained after sintering for 1800 s. The large pores were formed due to the infiltration of the Al–Cu melt into fine pores.
- The powder mixture with a low initial relative density of 50% was densified significantly at the early stage of sintering. In situ observation using optical microscopy revealed a significant rearrangement of Al powder at the early sintering stage, which promoted the densification.
- This study suggests that the liquid-phase sintering of Al powder using Al–Cu aids is suitable for BJT additive manufacturing, which requires the sintering of green bodies with a low relative density of 40–60%.

## Figures and Tables

**Table 1:** Experimental conditions for sintering tests and in situ observations in this study.

Ex Situ/In Situ	Methods	Conditions
Ex situ sintering tests	Sintering using an induction furnace Relative density measurements scanning electron microscopy (SEM)	Initial relative density: 50%, 90% Temperature: 630 °C Time: 0–1800 s Atmosphere: Ar (0.05 MPa)
In situ observations	Synchrotron X-ray computed tomography (CT)	Initial relative density: 90% Temperature: 660 °C Time: 0–1800 s Atmosphere: N_2_ flow
	Scanning electron microscopy (SEM)	Initial relative density: 90% Temperature: 630 °C Time: 0–1800 s Atmosphere: Ar (50 Pa)
	Optical microscopy (OM)	Initial relative density: 50% Temperature: 630 °C Time: 0–1800 s Atmosphere: Ar flow

## Data Availability

The original contributions presented in this study are included in the article and its Supplementary Materials. Further inquiries can be directed to the corresponding author.

## Supplementary Materials

The following supporting information can be downloaded at: https://www.mdpi.com/article/10.3390/ma18245499/s1. Video S1: Supplementary_material_1; Video S2: Supplementary_material_2.
